# 25-OH Vitamin D Levels and Cognitive Performance: Longitudinal Assessment in a Healthy Aging Cohort

**DOI:** 10.3389/fnagi.2019.00330

**Published:** 2019-11-27

**Authors:** André Couto Carvalho, Nadine Correia Santos, Carlos Portugal-Nunes, Teresa Costa Castanho, Pedro Moreira, Patrício Soares Costa, Nuno Sousa, Joana Almeida Palha

**Affiliations:** ^1^Life and Health Sciences Research Institute (ICVS), School of Medicine, University of Minho, Braga, Portugal; ^2^ICVS/3B’s, PT Government Associate Laboratory, Braga/Guimaraes, Portugal; ^3^Clinical Academic Center, Braga, Portugal; ^4^Division of Endocrinology, Diabetes and Metabolism, Santo Antonio Hospital—Centro Hospitalar Universitário do Porto, Porto, Portugal

**Keywords:** 25-hydroxyvitamin D, vitamin D, aging, healthy aging, cognitive performance, longitudinal analysis

## Abstract

**Background**: Declining serum levels of 25-hydroxyvitamin D [25(OH)D, a biomarker of vitamin D status] with aging is a well-recognized phenomenon. However, scarce information is available on the relation between 25(OH)D levels and cognitive performance over time in older individuals. Our purpose was to evaluate, longitudinally, the association of 25(OH)D with cognitive function in a healthy older adults’ cohort.

**Methods**: Sixty-four individuals over 55 years-old with no cognitive impairment, clustered as healthy “Poor” and “Good” cognitive performers, were followed for an average of 18 months. Seasonal-adjusted 25(OH)D serum levels (measured by high-performance liquid chromatography-tandem mass spectrometry) were related, longitudinally, with cognitive (memory and general/executive) composite scores.

**Results**: Overall seasonal-adjusted median serum 25(OH)D level was of 47 nmol/l [interquartile range (IQR), 38–60 nmol/l]. A negative correlation between baseline 25(OH)D and the general/executive composite score was found in the “Poor” cognitive performers (*r*^s^ = −0.52, *p* = 0.006), an association lost after adjusting 25(OH)D levels for the season. No effect was found in both groups between seasonal-adjusted 25(OH)D levels and the variation of both memory and general/executive composites during follow-up when adjusted for age, gender and education level.

**Conclusion**: In this healthy older population with no cognitive impairment, lower serum levels of 25(OH)D were not longitudinally associated with poorer cognitive scores.

## Introduction

Vitamin D is a steroid prohormone obtained from the diet or produced by the action of ultraviolet light in the skin (Bouillon, 2016). Once in circulation, it is rapidly hydroxylated in the liver into 25-OH vitamin D [25(OH)D]. Levels of 25(OH)D are considered surrogate indicators of vitamin D homeostasis (Bouillon, 2016). While its best well-known function resides on the regulation of calcium homeostasis, evidence is accumulating on the association of vitamin D deficiency with reduced musculoskeletal health and increased risk for acute and chronic diseases, as well as all-cause mortality (Pludowski et al., 2013; Schöttker et al., 2014). More so, some studies found significant positive associations between blood 25(OH)D and several cognitive performance scores in different gender and age groups (Annweiler et al., 2010; van der Schaft et al., 2013; Anastasiou et al., 2014; Granic et al., 2015). However, available observational data in older populations, evaluating various cognition domains and 25(OH)D levels provided contradictory results. Several, but not all, cross-sectional and prospective studies involving adults older than 60 years have shown an increased risk of cognitive impairment for those with low levels of 25(OH)D (Annweiler et al., 2013; Goodwill et al., 2018). Still, overall, very few studies have addressed this issue longitudinally in a community-dwelling senior population setting and with a standardized 25(OH)D serum measurement method (Perna et al., 2014; Kuźma et al., 2016).

Here the objective was to evaluate the association between cognitive longitudinal performance (average follow-up of 18 months) and 25(OH)D serum levels determined by high-performance liquid chromatography-tandem mass spectrometry. The population sample was comprised of healthy individuals aged 55 years and older, with no cognitive impairment, but characterized by distinct “normal” cognitive performance patterns.

## Materials and Methods

### Subjects

**Figure 1:**
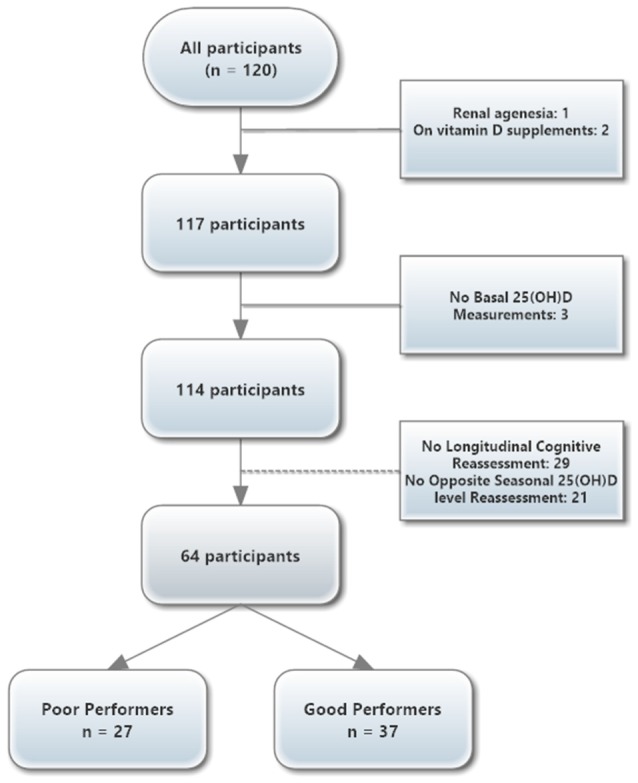
Overview of the participant flowchart.

The study was conducted between March 2012 and March 2015. Summarily, the recruitment was performed in two-phases. First, a larger sample, representative of the general Portuguese older population in terms of age, gender, and education underwent a full neuropsychological assessment (subjects were randomly selected from the Guimarães and Vizela local area health authority registries), resulting in 1,051 participants after inclusion/exclusion criteria (Costa et al., 2013; Santos et al., 2013, 2014). Then, of these, 120 subjects (matched for gender and age) were chosen in order to provide cognitive profiles of overall “good” cognitive performance (*n* = 60) and overall “poor” performance (*n* = 60) group, based on their, within normal range, neuropsychological testing. Primary exclusion criteria included inability to understand informed consent, participant choice to withdraw from the study, dementia and/or diagnosed neuropsychiatric and/or neurodegenerative disorder. Adjusted thresholds for cognitive impairment were calculated depending on factors such as age and/or education (Grigoletto et al., 1999; Busch and Chapin, 2008). Thus, the Mini-Mental State Examination (MMSE) test score thresholds applied were the following: MMSE score <17, if individual with 4 or less years of formal school education and/or 72 or more years of age, and MMSE score <23 otherwise (follows the MMSE validation study for the Portuguese population; Guerreiro et al., 1994). All participants were community dwellers. For final analysis purposes, subjects with prior history of renal failure, cerebrovascular disorders, osteomalacia, any other bone disease, or those who were on calcium and/or vitamin D supplements were excluded. Individuals who presented estimated glomerular filtration rate below 50 ml/min/1.73m^2^ and/or who did not have 25(OH)D serum concentration data available were also excluded. The final sample for consideration to the longitudinal analysis was of 64 participants, all of them attended the complete evaluation sessions and had a “seasonal-adjusted” vitamin D evaluation (Figure 1).

The study was conducted in accordance with the Declaration of Helsinki and approved by national and local ethics review boards. All study goals and nature of the tests were explained to the potential participants and informed signed consent obtained.

### Analytical Methods

The season of blood collection was dichotomized into Winter-Spring (between 21st December and 20th June) and Summer-Autumn (between 21st June and 20th December), based on the usual solar solstices/equinoxes’ dates. All subjects underwent fasting for morning blood collection. Blood samples were collected, centrifuged and stored at −20°C until 25(OH)D levels determination. A seasonal-adjusted mean 25(OH)D levels were obtained for each participant with two blood samples performed on a different season collection (Winter-Spring and Summer-Autumn) over a 1–2 years period follow-up to create an individual “season-adjusted” value (*n* = 64). The 25(OH)D measurement was obtained by a high-performance liquid chromatography-tandem mass spectrometry (LC-MS/MS) method (Waters Corporation, Milford, MA, USA) with a coefficient of variation of 4.9%. As expected (since individuals under supplementation were excluded from the study), 25(OH)D2 levels were negligible (corresponded to less than 1% of the full D2+D3 concentration). Therefore, total 25(OH)D levels reported here correspond to the 25(OH)D3 determination.

Vitamin D deficiency definition based on serum 25(OH)D values is not consensual. For description purposes, we categorized into three groups based on: 30 nmol/l (12 ng/ml), 50 nmol/l (20 ng/ml) and 75 nmol/l (30 ng/ml) 25(OH)D cut-point levels (“deficiency,” “adequacy” and “optimal” thresholds, respectively; Holick et al., 2011).

### Vitamin D Intake

Vitamin D intake was assessed by a 24 h diet recall questionnaire. This estimation was performed using the Nutrilog^®^ software (Nutrilog SAS, France), resorting to the release 23 of the United States Department of Agriculture National Nutrient Database for Standard Reference and adapted to the Portuguese foods using the Portuguese Food Composition Database (INRJ, 2018). Food vitamin D fortification is not commonly practiced in Portugal (Barroso, 2014).

### Cognitive Assessment

A team of trained psychologists performed the cognitive/neuropsychological assessments. A test battery was used for socio-demographic characterization and to evaluate multiple neuropsychological dimensions, including cognition profiles [general cognitive status and executive (EXEC) and memory (MEM) functions], as previously reported (Costa et al., 2013). Briefly, these included: Graffar socio-demographic scale, digit-span forward and backward test, Stroop color and word test, controlled oral word association test (COWAT), selective reminding test (SRT), digit symbol substitution test (DSST) and MMSE (scores adjusted for cognitive impairment and Portuguese population; Guerreiro et al., 1994). A Principal Component Analysis was performed in order to allocate the multiple test variables into composite components/dimensions, as previously reported (Santos et al., 2013). Summarily, this resulted in the identification of significant dimensions: memory (MEM; SRT test variables: consistent long-term retrieval, long-term storage and delayed recall) and general/executive function (EXEC; COWAT letters F-A-S admissible parameter; Stroop parameters: words, colors and words/colors, digits parameters: forward and backward; MMSE). A z-score for the cognitive composite was calculated and used to select normal extreme values and select “Poor” and “Good” cognitive performers. In our population, the suitability of using this battery of cognitive tests to measure two latent constructs, memory and executive functioning, was previously demonstrated by a longitudinal invariance analysis across the follow-up (Moreira et al., 2018).

### Statistical Analysis

All data are presented as the mean (median), standard deviation (SD); inter-quartile-range (IQR) for normally (non-normally) distributed data. All continuous variables were checked for normality using the Shapiro-Wilk normality test. Unpaired *t*-test and Mann-Whitney *U*-test were used to compare continuous variables between the two groups, as appropriate. Wilcoxon matched-pairs signed-rank test was performed to compare paired variables that failed normality tests. Fisher’s exact test was used for categorical variables. The confidence interval of a proportion was obtained by the modified Wald method. Univariate analysis with Spearman’s rank correlation was performed to assess linear relationship between 25(OH)D levels and the different cognitive domain scores (MEM and EXEC) and its temporal trends, stratified by cognitive group. A multiple linear regression analysis was conducted to explore the association between seasonal-adjusted 25(OH)D levels and age, gender and vitamin D intake estimation. Similarly, a multiple linear regression analysis was done to evaluate the prediction of Memory/Executive function scores over time by seasonal-adjusted 25(OH)D levels, adjusted to age, gender, baseline cognitive group and education level. Effect size estimates were calculated with Cohen’s *d* and *η*^2^ for continuous variables with parametric and non-parametric comparisons, respectively; *r* for Wilcoxon matched paired test; φ coefficient for Fisher’s exact test; and *R*^2^ and adjusted *R*^2^ for regression analysis.

All analyses were tested at the 0.05 level of significance and performed using IBM SPSS Statistics, v.21 (IBM, New York, NY, USA) and GraphPad Prism, v.6.00 (GraphPad Software, La Jolla, CA, USA). Effect size estimates were evaluated by Cohen’s published benchmark classes (small, medium and large) with the following proposed cut-points: 0.2, 0.5 and 0.8 for Cohen’s *d*; 0.01, 0.06, 0.14 for *η*^2^ 0.1, 0.3 and 0.5 for *r* and φ coefficient; and 0.02, 0.13, 0.26 for *R*^2^ (Cohen, 1988).

## Results

After exclusion criteria, the final sample included 64 individuals with a mean age of 65 years (SD 8 years), a women ratio of 0.47 (*n* = 30) and a median follow-up of 18 months (min–max: 16–22 months). Median 25(OH)D levels were higher during Summer-Fall when compared to Winter-Spring season (55 nmol/l vs. 43 nmol/l, *p* = 0.01, *η*^2^ = 0.06). The overall median seasonal-adjusted serum 25(OH)D level was 47 nmol/l (IQR 38–60 nmol/l; range 13–114 nmol/l). Total study population characteristics are presented in Table 1. Overall vitamin D deficiency (below 30 nmol/l) was found in 10 subjects (16%, 95% CI 9–27%), with only seven participants (11%, 95% CI 5–21%) surpassing the threshold of optimal 25(OH)D serum concentration (≥75 nmol/L). Thirty-eight participants (59%, 95% CI 47–71%) had 25(OH)D levels below 50 nmol/l. These proportions were not different between “Good” and “Poor” groups (Table 1). The estimated median vitamin D intake was 0.9 μg (36 IU) per day [IQR 0.2–3.1 μg (8–124 IU) per day], and not different among cognitive groups (1.5 *vs*. 0.5 μg/day, *p* = 0.06), with a moderate/low practical effect size (*η*^2^ = 0.06).

**Table 1:** Study population characteristics by cognitive performance group, *n* = 64.

	Total (*n* = 64)	“Poor” (*n* = 27)	“Good” (*n* = 37)	*p*	Effect size
Socio-demographic features					
Sex					
Female, *n* (%)	30 (47)	14 (52)	16 (43)	0.61	0.09
Age, mean (SD), years	65 (8)	67 (7)	64 (9)	0.09	0.04
Education level above 4-years, *n* (%)	17 (27)	2 (8)	15 (41)	0.004	0.37
Vitamin D parameters					
Seasonal-adjusted median serum 25(OH)D concentration, nmol/l (IQR)	47 (38–60)	47 (40–54)	47 (36–61)	0.82	0.001
Vitamin D status [serum 25(OH)D levels]					
Below 30 nmol/l (“deficient”), *n* (%)	10 (16)	3 (11)	7 (19)	0.50	0.11
Below 50 nmol/l (“inadequate”), *n* (%)	38 (59)	16 (59)	22 (60)	>0.99	0.002
Between 50 and 74 nmol/l (“adequate”), *n* (%)	19 (30)	9 (33)	10 (27)	0.60	0.07
75 nmol/l or above (“optimal”), *n* (%)	7 (11)	2 (7)	5 (14)	0.69	0.01
Vitamin D intake estimation, μg/day, median (IQR)*	0.9 (0.2–3.1)	0.5 (0.1–2.3)	1.5 (0.2–5.6)	0.06	0.06
Cognitive scores and trends, Median (IQR)					
Baseline MEM score	0.23 (−0.97 to 0.74)	−0.98 (−1.27 to −0.73)	0.62 (0.35–0.98)	<0.001	0.72
Baseline EXEC score	0.09 (−0.84 to 0.81)	−0.89 (−1.05 to −0.72)	0.70 (0.41–1.27)	<0.001	0.72
Last MEM score	−0.65 (−1.19 to 0.49)	−1.16 (−1.49 to −0.91)	0.09 (−0.65 to 1.04)	<0.001	0.35
Last EXEC score	−0.17 (−0.94 to 0.89)	−0.96 (−1.26 to −0.55)	0.793 (0.45–1.31)	<0.001	0.71
MEM score Trend	−0.30 (−0.71 to 0.13)	−0.19 (−0.45 to 0.19)	−0.44 (−1.23 to 0.11)	0.03	0.04
EXEC score Trend	−0.004 (−0.11 to 0.14)	−0.003 (−0.12 to 0.16)	−0.009 (−0.10 to 0.14)	0.69	0.003

IQR, interquartile range; MEM, memory dimension composite; EXEC, executive function dimension composite. *n = 63, 27 vs. 36.

On univariate analysis, “Good” performers had higher education (*p* = 0.004, φ = 0.37) when compared to “Poor” performers. Cognitive domain scores at baseline and final evaluation were different between the two groups (*p* < 0.001), with a high practical effect size (*η*^2^ = 0.72), for both domains. MEM score tends to decrease over time in both groups but reached statistical significance only in the “Good” group (*p* < 0.001), with a very high practical effect size (*r* = 0.56). EXEC scores were stable (“Poor”: *p* = 0.83, *r* = 0.04; “Good”: *p* = 0.53, *r* = 0.10) and not different between “Poor” and “Good” groups (*p* = 0.69, *η*^2^ = 0.003), with low practical effect size. There was no association between baseline 25(OH)D levels and MEM and EXEC scores (at baseline, last and longitudinal variation) in both groups, except for the first EXEC score obtained in the “Poor” performance group, with a high negative correlation score (*r*^s^ = −0.52, *p* = 0.006; Table 2). Seasonal-adjusted 25(OH)D levels were not correlated to any MEM and EXEC scores or to its longitudinal variation in both groups (Table 2).

**Table 2:** Spearman’s rank correlation (*r*^s^) between 25(OH)D levels and MEM and EXEC scores and its temporal trends (in both “Poor” and “Good” performers).

	“Poor” (*n* = 27)		“Good” (*n* = 37)	
	Baseline 25 (OH)D	Seasonal-adjusted 25 (OH)D	Baseline 25 (OH)D	Seasonal-adjusted 25 (OH)D
Baseline MEM score	−0.25	−0.06	0.31	0.25
Baseline EXEC score	−0.52^a^	−0.05	0.22	0.16
Last MEM score	−0.21	−0.05	0.20	0.16
Last EXEC score	−0.26	−0.01	0.11	0.08
MEM score trend	0.15	0.12	0.10	0.08
EXEC score trend	0.24	0.27	−0.13	−0.03

*^a^p = 0.006*.

To explore the association between the observed variations in MEM and EXEC scores over time with seasonal-adjusted 25(OH)D levels adjusted for baseline age, gender, cognitive performance and education level, a multiple linear regression analysis was performed (Table 3). In this fully adjusted model, 25(OH)D seasonal-adjusted serum concentration failed to predict cognitive performance over time (MEM score trend, *p* = 0.96, with moderate effect size: *R*^2^ = 0.19, adjusted *R*^2^ = 0.12; EXEC score trend, *p* = 0.47, with low effect size: *R*^2^ = 0.07, adjusted *R*^2^ = −0.005). Being a “Poor” performance individual was linked to a less detrimental decay in MEM score over time (*β* = 0.68, 95% CI 0.29–1.08, *p* = 0.001). No other variable evaluated showed a relevant effect on MEM or EXEC score trends (Table 3).

**Table 3:** Association between observed Memory/Executive Function scores trend with age, gender, cognitive group, education level and “seasonal-adjusted” 25(OH)D levels predicted by multiple linear regression analysis (*n* = 64; MEM score trend, *p* = 0.96, *R*^2^ = 0.19, adjusted *R*^2^ = 0.12; EXEC score trend, *p* = 0.47, *R*^2^ = 0.07, adjusted *R*^2^ = −0.005).

	MEM score trend		EXEC score trend	
	*β* (95% CI)	*p*	*β* (95% CI)	*p*
Age (years)	−0.01 (−1.95 to 1.82)	0.29	−0.002 (−0.01 to 0.006)	0.60
Gender (Exposure: Male)	−0.04 (−0.43 to 0.35)	0.84	−0.07 (−0.12 to 0.05)	0.25
Cognitive performance (Exposure: “Poor”)	0.68 (0.29–1.08)	0.001	−0.01 (−0.14 to 0.11)	0.84
Education Level (years)	0.05 (−0.004 to 0.11)	0.07	0.01 (−0.005 to 0.03)	0.15
Seasonal-adjusted 25(OH)D levels (nmol/l)	0.001 (−0.01 to 0.01)	0.96	−0.001 (−0.004 to 0.002)	0.47

## Discussion

In this longitudinal study with a healthy non-demented older cohort, we found no association between standardized season-adjusted 25(OH)D levels and the variation of the “memory” and “executive” cognitive domains over a median follow-up of 18 months.

An important seasonal effect over cognitive performance is currently recognized, with annual variations in brain function reaching a peak in late Summer and early Fall and declining in late Winter and early Spring (Meyer et al., 2016; Lim et al., 2018). This observation may help explain some intraindividual cognitive changes that develop at specific times of the year and the importance of seasonal adjusting when exploring cognitive associations with other variables that share circannual cycles. We observed a negative correlation between baseline 25(OH)D values and baseline EXEC score in the “Poor” performers group, but this effect was lost after adjusting 25(OH)D for season. Our observations are in accordance with the few studies that evaluated cognitive performance and behavior in populations exclusively with vitamin D insufficiency like ours. Some authors using a very low 25(OH)D cut-point level (25 nmol/l), have shown a small or no clear positive association with cognitive performance (Aung et al., 2006; Annweiler et al., 2010). Others, using a somewhat higher 25(OH)D threshold levels, have reported better cognitive performance associated with these “healthier” vitamin D levels (Llewellyn et al., 2011; Annweiler and Beauchet, 2014). Recently, a similar study with non-demented German older adults also found that the lowest 25(OH)D quintiles were associated with higher cognitive decline (based on a Cognitive Telephone screening instrument score—COGTEL) over an average 5-year follow-up time (Perna et al., 2014). Interestingly, one study reported that this “positive” effect of 25(OH)D concentrations and cognition tests were only present in the sub-group with low number of years of formal education (Assmann et al., 2015). In the current study, despite the overall low level of standard school education years, no such relationship was observed. Further studies are required to clarify whether there is a minimal 25(OH)D threshold that provides neuroprotection in still cognitive healthy populations.

Our multivariate analysis reveals no effect of 25(OH)D on the temporal trends observed in the MEM and EXEC composites in both groups. With this regard, it is important to consider that in contrast to most studies reporting association with single neuropsychological tests, here multiple cognitive components were characterized and grouped into a comprehensive neuropsychological domain composite. When considered alone, the various studies performed to date were seldom concordant. Two recent meta-analyses, including 14 longitudinal studies, indicate that lower 25(OH)D levels are indeed associated with some executive dysfunctions (especially on mental shifting, information updating and processing speed) and cognitive decline. However, they do not provide clear evidence for the relation of 25(OH)D levels with episodic memory or whether there is a 25(OH)D threshold/optimal therapeutic window for supplementation to prevent cognitive decline later in life (Annweiler et al., 2013; Goodwill and Szoeke, 2017). These same inconsistencies have been reported more recently in larger and longer prospective cohorts. In a large study spanning over 20-years, 25(OH)D levels measured in midlife were not associated with more rapid cognitive decline over the follow-up period (Schneider et al., 2018). However, another large US-based cohort submitted to extensive neuro-cognitive tests covering several domains found that after a mean 5-year follow-up, higher baseline serum 25(OH)D concentrations were linked to a slower rate of decline in a test of verbal fluency (Beydoun et al., 2018). Moreover, a very recent study failed to observe any relationship between baseline 25(OH)D levels and cognition or cognitive decline over 2 years of follow-up by evaluating 1499 Puerto Rican participants living in the Boston Area (aged 45–75 years old at baseline) and using a principal-components analysis to quantify the association between 25(OH)D and the longitudinal performance of two major cognitive features: executive function, and memory (Palacios et al., 2019).

Unfortunately, current published intervention trials addressing vitamin D and cognition have not provided a conclusive answer (Dhesi et al., 2004; Przybelski et al., 2008; Dean et al., 2011; Stein et al., 2011; Rossom et al., 2012; Pettersen, 2017; Rutjes et al., 2018; SanMartin et al., 2018; Castle et al., 2019). A recent small intervention trial showed general cognitive status improvement in 16 mild cognitive impaired patients after 18 months of vitamin D supplementation (but not in healthy controls or those already diagnosed with dementia; SanMartin et al., 2018). Improved visual memory was observed in healthy Canadian adults with “insufficient” baseline 25(OH)D levels (below 75 nmol/l) that were supplemented with high doses of vitamin D (4,000 IU per day) for 18 weeks, without replication in other cognitive domains (Pettersen, 2017). More recently, in a randomized controlled trial with 69 overweight/obese postmenopausal women with 25(OH)D levels less than 75 nmol/l and divided in three different intervention doses of vitamin D (600, 2,000 or 4,000 IU per day), only the intermediate group performed better in learning and memory tests after 1 year supplementation (Castle et al., 2019). These results reflect the complexity behind any potential vitamin D effect on cognition and the need for better-designed studies, probably including distinct vitamin D insufficiency status populations, supplement doses, exposures periods, age groups and baseline cognitive performances.

Another important observation of our study pertains with the 25(OH)D status of the individuals in the study population. Levels below 30 nmol/l were found in 16% and below 50 nmol/l in almost 60%. These results provide a less dramatic indication of vitamin D deficiency/inadequacy compared to recently published data on the Portuguese population (levels below 30/50 nmol/l were detected in 40/69% of a nationwide cluster sample of 1,500 Portuguese subjects over 65 years old or 38/86% of a subsample of another national cross-sectional study that included Portuguese adults registered in mainland primary health care centers; Raposo et al., 2017; Santos et al., 2017). Much of the differences reported in 25(OH)D levels are probably based on the season-geography confounder and on methodological procedures used (Cashman et al., 2016). In the present study, the LC-MS/MS was used, which provides a more accurate measure of vitamin D status when comparing to some immunological methods used in routine determinations, and this technicality is likely to justify the main differences found between this and other studies coming from similar Portuguese populations (Wallace et al., 2010).

Self-reported daily intake of vitamin D data deserves consideration. Despite the limitation of self-reported data, the estimated median vitamin D intake was only of 0.9 μg/day (36 IU/day; IQR 0.2–3.1 μg/day, 8–124 IU/day), with all subjects evaluated failing to achieve the recommended vitamin D intake (15 μg/day for adults with less than 70 years and 20 μg/day for those older than 70 years; Aloia, 2011). Main dietary sources of vitamin D are meat, fish, milk and cheese. Overall, in our cohort, meat consumption (meat/fish or eggs was present in 54% of the meals with no individuals reporting being vegetarian—data not shown) was relatively small (mean of 145 mg per meal—data not shown). The current Portuguese Food Composition Database states that eggs and meat have generally low levels of vitamin D (between 0.7–1.7 μg per 100 mg; INRJ, 2018). Vitamin D supplementation in food is not mandatory in Portugal and most “supplemented” dairy products present levels of vitamin D between 0.3 and 1.5 μg per 100 ml or 100 g (Parreira et al., 2015). These two facts may imply that, in our sample, even an “usual” daily consumption of 200 g of meat/eggs or “supplemented” dairy products, would represent no more than 20% of the recommended intake of vitamin D. Current habitual intakes of vitamin D in most other countries are also low (typically around 1–4 μg/day), meaning that the proposed reference intake value has to be covered mostly by additional vitamin D supplements and/or endogenous synthesis (Brown et al., 2013). This observation suggests that circulating vitamin D levels in these individuals is most likely provided by exposure to sun. It is important to recall that the present study focuses on a population of active healthy individuals living in the community and that despite the tendency observed towards higher vitamin D intake in the “Good” performing group, no such reflection was present in their circulating 25(OH)D levels.

The study main strengths are grounded on the exhaustive cognitive evaluation performed longitudinally with the construction of coherent executive and memory cognitive domain composites and a two-point season-opposite 25(OH)D levels determination. However, some important limitations of the study should be considered. First, there was a relatively small number of participants available for final exploration with a complete longitudinal cognitive assessment, which decreased the sensitivity to detect any small effect and prevented further analysis comparing distinct groups based on more extreme 25(OH)D distribution. Second, there was a relatively short time between the two cognitive evaluations (min-max: 16–22 months), which may have also impaired the ability to detect subtle intra or inter-individual cognitive time-dependent differences, underestimating potential associations with 25(OH)D levels. Third, despite the two seasonal opposite 25(OH)D determinations separated by at least 1.5-years, no “real” prolonged longitudinal evaluation of 25(OH)D levels was made. This means that individual vitamin D status appraisal may have resulted in a rather 2-year “cross-sectional” estimation and prevented, therefore, any assessment of its expanded longitudinal effect on cognition. Also relevant is the fact that the study population was almost homogenously vitamin D insufficient. This feature, frequently found in other studies with older adults, makes it harder to discover any associations given the lack of 25(OH)D levels inter-individual variability found in these cohorts (Annweiler et al., 2011).

In summary, in this Portuguese older healthy population, serum 25(OH)D levels were not associated with cognitive domain composite scores for an average 18 months follow-up, in both stronger and poorer cognitive performers. Despite the lack of agreement about what should be the optimal circulating 25(OH)D levels, the conclusions of the present study are limited to the neurocognitive outcomes obtained in older individuals with relatively stable low 25(OH)D levels and during a short longitudinal period. Further studies should address the role of vitamin D during several brain development time frames and its role in cognitive aging.

## Ethics Statement

This study was performed in accordance with the Declaration of Helsinki and approved by national and local ethics review boards (Hospital de Braga, Centro Hospitalar do Alto Ave and Unidade Local de Saúde do Alto Minho) and by the national data protection entity (Comissão Nacional de Protecção de Dados). All study goals and nature of the tests were explained to the potential participants and informed signed consent obtained.

## Author Contributions

AC, NCS, NS and JP designed the study protocol. AC, NCS, CP-N, TC and PM collected the data. Data analysis and interpretation by AC, NCS and PC. AC wrote the first draft under supervision of JP. AC, NCS, CP-N, TC, PM, PC, NS and JP contributed significantly to revising the manuscript. All authors read and approved the final manuscript.

## Conflict of Interest

The authors declare that the research was conducted in the absence of any commercial or financial relationships that could be construed as a potential conflict of interest.
